# A meta-analysis of survival after minimally invasive radical hysterectomy versus abdominal radical hysterectomy in cervical cancer: center-associated factors matter

**DOI:** 10.1007/s00404-021-06348-5

**Published:** 2022-01-21

**Authors:** Si Sun, Jing Cai, Ruixie Li, Yujia Wang, Jing Zhao, Yuhui Huang, Linjuan Xu, Qiang Yang, Zehua Wang

**Affiliations:** Department of Obstetrics and Gynecology, Union Hospital, Tongji Medical College, Huazhong University of Science and Technology, Wuhan, 430022 China

**Keywords:** Cervical cancer, Minimally invasive surgery, Laparoscopic radical hysterectomy, Robotic-assisted radical hysterectomy, Abdominal radical hysterectomy, Oncological outcome, Overall survival, Disease-free survival, Recurrence

## Abstract

**Purpose:**

To explore the possible factors that contributed to the poor performance of minimally invasive surgery (MIS) versus abdominal surgery regarding progression-free survival (PFS) and overall survival (OS) in cervical cancer.

**Methods:**

MEDLINE, EMBASE, Cochrane Library and Web of Science were searched (January 2000 to April 2021). Study selection was performed by two researchers to include studies reported oncological safety. Summary hazard ratios (HRs) and 95% confidence intervals (CIs) were combined using random-effect model. Subgroup analyses were stratified by characteristics of disease, publication, study design and treatment center.

**Results:**

Sixty-one studies with 63,369 patients (MIS 26956 and ARH 36,049) were included. The overall-analysis revealed a higher risk of recurrence (HR 1.209; 95% CI 1.102–1.327) and death (HR 1.124; 95% CI 1.013–1.248) after MIS versus ARH expect in FIGO IB1 (FIGO 2009 staging) patients with tumor size less than 2 cm. However, subgroup analyses showed comparable PFS/DFS and OS in studies published before the Laparoscopic Approach to Cervical Cancer (LACC) trial, published in European journals, conducted in a single center, performed in centers in Europe and in centers with high sample volume or high MIS sample volume.

**Conclusion:**

Our findings highlight possible factors that associated with inferior survival after MIS in cervical cancer including publication characteristics, center-geography and sample volume. Center associated factors were needed to be taken into consideration when evaluating complex surgical procedures like radical hysterectomy.

**Supplementary Information:**

The online version contains supplementary material available at 10.1007/s00404-021-06348-5.

## Introduction

Minimally invasive surgery (MIS), including laparoscopic radical hysterectomy (LRH) and robotic-assisted radical hysterectomy (RRH), had long been recognized as an alternative surgical approach to abdominal radical hysterectomy (ARH) with reduced operative morbidity and similar oncological safety until 2018 [1, 2]. Either the preliminary data that drew an early end to a randomized controlled trial (the laparoscopic approach to cervical cancer, LACC) or the result of a large-scale observational study revealed inferiority of overall survival (OS), disease-free survival (DFS) and progression-free survival (PFS) in patients undergoing MIS compared to ARH. These results shattered the long standing consensus of preference of MIS as primary treatment for cervical cancer and the clinical practice guideline in cervical cancer changed accordingly [3].

Before LACC, most studies compared MIS versus ARH in cervical cancer reported that MIS showed better short-term outcomes and equivalent 5-year survival compared to ARH [4–8], hence three meta-analyses based on data before LACC reporting that there was no difference of risk of recurrence or death between patient underwent MIS and ARH [9, 10]. After LACC, evidence implying inferiority of MIS for managing cervical cancer sprouted and mounted [11–14].Three recent meta-analyses evaluated the issue on basis of different inclusion criteria and ended up with different conclusions: Tanitra et al. included five studies published before 2018 and suggested no difference of PFS or OS between MIS and ARH; Nitecki et al. identified 49 studies and included 15 high-quality studies in their meta-analysis comparing MIS and ARH in patients with FIGO IA1 to IIA (FIGO 2009 staging) cervical cancer suggesting that MIS was associated with increased risks of both recurrence and death; Hwang et al. included 36 studies comparing DFS of patients undergoing LRH and ARH suggested that LRH was associated with higher risk of recurrence in patients with tumor size larger than 2 cm [15–17].

MIS revealed superiority of survival outcomes in prostate, colon and rectum cancers and equivalent outcomes in endometrial cancer according to the Gynecologic Oncology Group Study LAP2 trial and the laparoscopic approach to cancer of the endometrium LACE trial [18–20]. Researchers proposed several hypothetical factors that might lead to poor performance of MIS in cervical cancer such as uterine manipulator, CO_2_ pneumoperitoneum, learning curve, hospital volume, technique of surgeons and tumor size in patients [21, 22]. Therefore, we aim to evaluate the oncological safety of MIS in cervical cancer patients stratified by characteristics of disease (FIGO stage and tumor size), publication (publication time and journal), study design (single-center or multi-center) and treatment center (average sample size per center) and to identify possible factors that led to the controversies of MIS among previous studies.

## Method

### Literature search

The literature search was conducted in Medline, Embase, Pubmed, Cochrane library and Web of Science from January 2000 to April 2021 without limitation of text availability, article type or language using the following terms: “open”, “abdominal”, “laparotomy”, “laparoscopic”, “minimally invasive”, “robotic assisted”, “radical hysterectomy”, “surgery”, “cervical cancer”, “cervical carcinoma” and “carcinoma of the cervix”. Additional manual search was performed by scanning the references of all included and relevant studies.

### Study selection and quality assessment

Two authors screened the titles and abstracts for potentially related articles, which were further reviewed for eligibility by reference to the inclusion criteria as follows: (1) cervical cancer patients treated with minimally invasive or abdominal radical hysterectomy; (2) studies with at least two arms that compared OS, PFS or DFS; (3) patients that received surgery as primary treatment. Studies were excluded when (1) studies were published as comment, conference abstract and letter; (2) total number of patients less than 40 or at least one arm is less than 20; (3) studies did not provide sufficient data to estimate the hazard ratio (HR) and 95% confidence interval (CI) of OS, PFS or DFS between MIS and ARH; (4) patients that received radical trachelectomy or laparoscopic assisted radical vaginal hysterectomy; (5) patients received neoadjuvant radiotherapy or chemotherapy. When population overlap existed between studies, only the most recent published study with bigger population was included. Quality assessment was conducted using the Newcastle–Ottawa Scale (NOS) for assessing the quality of nonrandomized studies in meta-analysis (Supplemental file 1).

### Data extraction and subgroup classification

Two authors extracted the following data: name of first author, year of publication, journal of publication, region of journal, country and region where the studies were conducted, data source, number of centers, time span of enrollment, surgical approach, study type, cohort matching status, technique level of the surgeon, FIGO stage, histology, tumor size, lymphatic metastasis, adjuvant therapy, sample size before and after propensity score matching, HRs and 95% CIs of OS, PFS or DFS. HRs were estimated according to Tierney et al. if not reported [23]. The extracted data were validated by a third author. Since all included studies uniformly follow the 2009 FIGO staging criteria, the FIGO classification used in this study still represented the old nomenclature.

The subgroup classification criteria were as follows: (1) FIGO stage and tumor size, studies reporting patients with FIGO stage IB1 cervical cancer were classified into tumor size < 2 cm or ≥ 2 cm subgroup; (2) year of publication, studies were classified into published before or after the LACC trial subgroup; (3) region of journal, studies were classified according to the region of journal the studies were published; (4) number of centers, studies were classified into the single-center or the multi-center group; (5) surgical approach, studies were classified into the LRH vs. ARH, the RRH vs. ARH or the MIS vs. ARH subgroup; (6) region of center, studies were classified into different regional subgroups according to the geographical continental location of where the surgeries were conducted; (7) sample volume, sample volume referred to annual number of radical hysterectomies conducted by all means per center reported by each study, which was estimated by number of patients that received radical hysterectomy by all means before propensity scored matching dividing number of centers then dividing number of years of recruitment. Studies were classified into high sample volume group or low sample volume group by the cur-off of median value; (8) MIS sample volume, MIS sample volume referred to annual number of radical hysterectomies conducted by MIS per center reported by each study, which was estimated by number of patients that received radical hysterectomy by MIS before propensity scored matching dividing number of centers then dividing number of years of recruitment. Studies were classified into high MIS sample volume and low MIS sample volume group by the cur-off of median value.

### Statistical analysis

Random-effect model was used for all analyses despite heterogeneity [24]. Adjusted HRs and HRs after propensity-scored matching were used for pooled analysis when applicable. Sample size before propensity-scored matching was used as the weight variance during meta-analysis. Heterogeneity of the included studies was assessed by *I*^2^ and *p* value according to Higgins et al. and was classified as small to modest (*I*^2^ < 50%) and high (*I*^2^ ≥ 50%) [25]. Publication bias was assessed by funnel plot and eager’s test. A 95% CI of HR not overlapping with 1 and a *p* value < 0.05 (two sided) were considered of statistical significance. All analyses were performed using STATA14 (MP-Parallel Edition, College Station, TX 77845 USA).

## Results

### Study characteristics

**Fig. 1:**
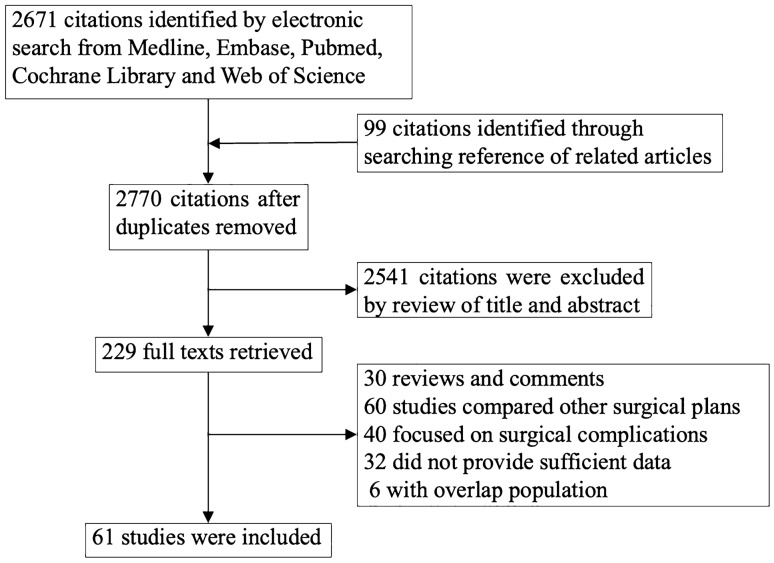
Flow chart of study search and selection

A total of 2770 citations were identified by electronic search (2671) and additional manual search (99) after removal of duplicates. 2541 were excluded by review of title and abstract. Full texts of the remaining 229 items were retrieved, of which 30 studies were reviews and comments, 60 studies compared the feasibility of different surgical plans, 40 focused on surgical complications, 32 studies did not provide sufficient data and 6 studies with smaller cohorts contained overlapping population. Finally, 61 eligible studies with 63,369 patients (MIS 26956, ARH 36049) were identified (Fig. 1). Basic characteristics of included studies were presented in Table 1. HRs and 95% CIs of DFS/PFS and OS were extracted and pooled from 58 studies (MIS 17092, ARH 14584) and 47 studies (MIS 17979, ARH 15493), respectively [1, 2, 4–8, 11–14, 18, 22, 26–73]. Comprehensive original data were shown in Supplemental file 2.

**Table 1:** Basic characteristics of included studies

Author	Year	Region^1^	Surgical approach	FIGO stage^3^	Cohort matching	No. of patients		Survival outcome
						MIS^4^	ARH	
Gennari P	2021	Europe	MIS vs ARH	IA to IIB2	Matched	302	111	OS, DFS
Kim S	2021	Asia	MIS vs ARH	IB1 to IIA2	Unmatched	110	38	OS, DFS
Levine M	2021	America	MIS vs ARH	IA1 to IB1	Unmatched	82	44	OS, DFS
Li L	2021	Asia	MIS vs ARH	IA to IIA	Unmatched	282	280	OS, DFS
Rodriguez J	2021	Mixed^2^	LRH vs ARH	IA2 IB1	Matched	681	698	OS, DFS
Zaccarini F	2021	Europe	LRH vs ARH	IA to IIIA	Matched	223	41	OS, DFS
Bogani G	2020	Europe	LRH vs ARH	IB1 to IIB	Unmatched	235	823	DFS
Brandt B	2020	America	MIS vs ARH	IA1 to IB1	Matched	117	79	OS, DFS
Chen B	2020	Asia	RRH vs ARH	IA1 to IIA2	Matched	1048	9266	OS, DFS
Chen C	2020	Asia	LRH vs ARH	IB1 ≤ 2 cm	Unmatched	963	1634	OS, DFS
Chen X	2020	Asia	LRH vs ARH	IB1 ≤ 2 cm	Unmatched	129	196	DFS
Chiva L	2020	Europe	MIS vs ARH	IB1	Matched	291	402	OS, DFS
Dai D	2020	Asia	LRH vs ARH	IB	Matched	213	213	OS, DFS
Eoh K	2020	Asia	RRH vs ARH	IA to IB	Matched	168	142	DFS
Guo C	2020	Asia	MIS vs ARH	IA1 to IIA1	Matched	2439	813	OS, DFS
He J	2020	Asia	LRH vs ARH	IA1 to IB1	Matched	2915	5545	OS, DFS
Hu T	2020	Asia	LRH vs ARH	IA2, IB1, IIA1	Matched	406	406	OS, DFS
Pedone L	2020	Europe	LRH vs ARH	IA1 to IIA1	Matched	137	114	DFS
Qin M	2020	Asia	LRH vs ARH	IA1 to IB1	Unmatched	172	84	OS, DFS
Uppal S	2020	America	MIS vs ARH	IA1 to IB1	Matched	560	255	OS, DFS
Wenzel H	2020	Europe	MIS vs ARH	IA2, IB1, IIA1	Matched	369	740	OS, DFS
Yang J	2020	America	RRH vs ARH	IA2 to IIA	Matched	152	181	OS, DFS
Yang W	2020	Asia	LRH vs ARH	IA IB1 IIA1	Matched	142	186	OS, DFS
Yuce T	2020	America	MIS vs ARH	IA to IB	Matched	1993	1707	OS
Alfonzo E	2019	Europe	RRH vs ARH	IA1 to IB1	Unmatched	628	236	OS, DFS
Cusimano M	2019	America	MIS vs ARH	IA, IB, II+	Unmatched	256	278	OS, DFS
Doo D	2019	America	RRH vs ARH	IB1	Unmatched	49	56	OS, DFS
Gil-Moreno A	2019	Europe	MIS vs ARH	IA2, IB1, IIA1	Unmatched	112	76	OS, DFS
Hu T	2019	Asia	LRH vs ARH	IA to IIA	Unmatched	255	423	DFS
Kanao H	2019	Asia	LRH vs ARH	IB1 to IIB	Matched	80	83	OS
Kim J	2019	Asia	LRH vs ARH	–	Matched	3100	3235	OS, DFS
Kim S	2019	Asia	LRH vs ARH	IB1 to IB2	Unmatched	158	435	OS, DFS
Liu Y	2019	Asia	LRH vs ARH	IB	Unmatched	271	135	OS, DFS
Matanes E	2019	Europe	RRH vs ARH	IA1 to IIB	Matched	74	24	OS, DFS
Paik E	2019	Asia	LRH vs ARH	IB1 to IIA1	Matched	119	357	OS, DFS
Ratiu D	2019	Europe	LRH vs ARH	IA1 to IIB	Matched	34	41	OS, DFS
Wang W	2019	Asia	LRH vs ARH	IB2 to IIB	Unmatched	231	197	OS, DFS
Yuan Z	2019	Asia	LRH vs ARH	IA2 to IIA2	Unmatched	98	98	OS
Alexander M	2018	America	MIS vs ARH	IA to IB	Matched	1225	1236	OS, DFS
Corrado G	2018	Europe	LRH vs ARH RRH vs ARH	IB1	Unmatched	152	101	DFS
Guo J	2018	Asia	LRH vs ARH	IA1 to IIA2	Unmatched	412	139	OS, DFS
Pedro R	2018	Mixed^2^	MIS vs ARH	IA1 to IB1	Unmatched	319	312	DFS
Driver	2017	America	MIS vs ARH	IA to IIB	Unmatched	101	282	OS, DFS
He H	2017	Asia	LRH vs ARH	IA2 to IIA2	Unmatched	1071	792	OS, DFS
Shah C	2017	America	RRH vs ARH	IA1 to IB2	Matched	107	202	DFS
Wallin E	2017	Europe	RRH vs ARH	IA1 to IIA2	Unmatched	149	155	OS, DFS
Mendivil A	2016	America	LRH vs ARH RRH vs ARH	IA2 to IIB	Unmatched	49	39	OS, DFS
Sert B	2016	America	RRH vs ARH	IA1 to IB2 +	Unmatched	259	232	OS, DFS
Wang W	2016	Asia	LRH vs ARH	IA2 to IIA2	Unmatched	203	203	OS, DFS
Zanagnolo V	2016	Europe	RRH vs ARH	IA2 to IIA2	Unmatched	203	104	DFS
Ditto A	2015	Europe	LRH vs ARH	IA2 IB1	Matched	60	60	OS, DFS
Xiao M	2015	Asia	LRH vs ARH	IA2 to IIB	Matched	106	48	OS, DFS
Yang L	2015	Asia	LRH vs ARH	IA2 to IIB	Unmatched	1052	477	OS, DFS
Bogani G	2014	Europe	LRH vs ARH	IA2 to IIB	Unmatched	65	65	OS, DFS
Toptas T	2014	Europe	LRH vs ARH	IA2 to IB1	Unmatched	22	46	OS, DFS
Ghezzi F	2013	Europe	LRH vs ARH	IB1 to IIB	Unmatched	68	273	DFS
Park J	2013	Asia	LRH vs ARH	IB2 and IIA2	Matched	115	188	OS, DFS
Choi C	2012	Asia	LRH vs ARH	IA1-IIA	Matched	105	99	DFS
Nam J	2012	Asia	LRH vs ARH	IA2 to IIA2	Unmatched	263	263	OS, DFS
Lee E	2011	Asia	LRH vs ARH	IA1 to IIB	Unmatched	24	48	OS, DFS
Yang Z	2011	Asia	LRH vs ARH	IA2 to IIA2	Matched	85	85	DFS
Cantrell L	2010	America	RRH vs ARH	IA1 to IIB	Matched	63	63	DFS
Malzoni M	2009	Europe	LRH vs ARH	IA2 IB1	Matched	65	62	DFS
Sobiczewski P	2009	Europe	LRH vs ARH	IA, IB1, IIA	Matched	22	58	OS, DFS
Li G	2007	Asia	LRH vs ARH	IB1 to IIA1	Matched	90	35	OS, DFS

*MIS* minimally invasive surgery, *LRH* laparoscopic radical hysterectomy, *RRH* robotic-assisted radical hysterectomy, *ARH* abdominal radical hysterectomy, *OS* overall survival, *DFS* disease-free survival

^1^Region of centers where the studies were conducted

^2^Mixed referred to intercontinental

^3^FIGO 2009 staging, FIGO IA1 specifically referred to those IA1 with LVSI

^4^MIS included LRH and RRH

### Meta-analyses of MIS versus ARH

Fifty-eight studies were included for meta-analysis of DFS/PFS comparing MIS to ARH. Total number of patient before propensity scored matching was 50,606 (MIS 20550 and ARH 29951) and 31,676 patients (MIS 17092 and ARH 14584) after matching were included for meta-analysis. The overall analysis revealed that patients who received MIS had higher risk of recurrence than patients that received ARH (HR 1.209; 95% C: 1.102–1.327). However, stratified analyses showed comparable PFS/DFS between the MIS and ARH patients in studies published before the LACC trial (HR 0.906; 95% CI 0.667–1.231), published in European Journals (HR, 0.919; 95% CI 0.717–1.178), conducted in a single center (HR 0.929; 95% CI 0.736–1.173), performed in centers in Europe (HR 1.027; 95% CI 0.789–1.338) or with high MIS sample volume (HR 1.028; 95% CI 0.857–1.234) (Table 2).

**Table 2:** Sub-group analyses of all studies comparing disease-free survival/progression-free survival between patients undergoing MIS and ARH

	No. of studies	No. of patients			HR (95% CI)	*p* value	*I*^2^ value
		MIS	ARH	Total			
Overall analysis	58	17,092	14,584	31,676	**1.209 (1.102–1.327)**	** < 0.001**	**54.0%**
Publication time-point							
Before LACC	23	4753	3951	8704	0.906 (0.667–1.231)	0.529	44.5%
After LACC	35	12,339	10,633	22,972	**1.294 (1.184–1.415)**	** < 0.001**	**59.8%**
Region of journal^1^							
Europe	16	2924	2552	5476	0.919 (0.717–1.178)	0.505	**65.1%**
America	32	10,161	8723	18,884	**1.254 (1.129–1.393)**	** < 0.001**	**55.8%**
Asia	9	3897	3271	7168	**1.421 (1.057–1.909)**	**0.020**	43.2%
No. of center							
Single center	33	12,926	11,097	24,023	0.929 (0.736–1.173)	0.535	32.7%
Multi-center	25	4166	3487	7653	**1.329 (1.209–1.461)**	** < 0.001**	**68.7%**
Surgical approach^2^							
LRH vs ARH	34	9083	8215	17,298	**1.193 (1.042–1.366)**	**0.011**	48.1%
RRH vs ARH	13	2333	2271	4604	**1.247 (1.065–1.460)**	**0.006**	**60.2%**
LRH&RRH vs ARH	13	9774	5676	4098	1.193 (0.966–1.474)	0.101	**64.3%**
Region of center^3^							
Europe	18	5189	2454	2735	1.027 (0.789–1.338)	0.843	**61.0%**
America	11	3749	1928	1821	**1.428 (1.045–1.950)**	**0.025**	48.1%
Asia	27	20,728	11,710	9018	**1.213 (1.092–1.347)**	** < 0.001**	43.0%
Sample volume^4^							
High volume	29	17,521	10,288	7233	**1.144 (1.012–1.294)**	**0.032**	**62.0%**
Low volume	27	12,353	6144	6209	**1.289 (1.112–1.493)**	**0.001**	41.7%
Mixed volume	2	1802	660	1142	**1.679 (1.196–2.358)**	**0.003**	**69.1%**
MIS sample volume^4^							
High volume	28	15,024	9229	5795	1.028 (0.857–1.234)	0.765	**56.4%**
Low volume	28	14,850	7203	7647	**1.319 (1.188–1.464)**	** < 0.001**	49.7%
Mixed volume	2	1802	660	1142	**1.679 (1.196–2.358)**	**0.003**	**69.1%**

Data in bold style were of statistical significance to faciliate reading

*MIS* minimally invasive surgery, *LRH* laparoscopic radical hysterectomy, *RRH* robotic-assisted radical hysterectomy, *ARH* abdominal radical hysterectomy, *HR* hazard ratio, *CI* confidence interval

^1^One study published in an Australian journal was not presented in the table

^2^Two studies reported both LRH and RRH

^3^Two studies conducted intercontinental were not presented in the table

^4^Sample volume and MIS sample volume were estimated by total number of patients received  radical hysterectomy by all means and by MIS before propensity scored matching dividing number of centers and years of recruitment

Forty-eight studies were included for meta-analysis of OS comparing MIS to ARH. Total number of patient before propensity scored matching was 59,212 (MIS 25347, ARH 33865) and 39,809 patients (MIS 21145 and ARH 18664) after matching were included for meta-analysis. Patients that received MIS had higher risk of death than patients who received ARH (HR 1.124; 95% CI 1.013–1.248). However, there were comparable OS between patients underwent MIS and ARH in studies published before the LACC trial (HR 0.857; 95% CI 0.628–1.169), published in European journals (HR 1.211; 95% CI 0.922–1.589), conducted in a single center (HR 1.021; 95% CI 0.772–1.352), performed in centers in Europe (HR 1.016; 95% CI 0.710–1.452) or Asia (HR, 1.028; 95% CI 0.906–1.166) and with a high MIS sample volume (HR 1.016; 95% CI 0.843–1.225). (Table 3).

**Table 3:** Sub-group analyses of all studies comparing overall survival between patients undergoing MIS and ARH

	No. of studies	No. of patients			HR (95% CI)	*p* value	*I*^2^ value
		MIS	ARH	Total			
Overall analysis	48	21,145	18,664	39,809	**1.124 (1.013–1.248)**	**0.028**	**66.4%**
Publication time-point							
Before LACC	15	3815	3048	6863	0.857 (0.628**–**1.169**)**	0.329	0.0%
After LACC	33	17,330	15,616	32,946	**1.175 (1.051–1.312)**	**0.004**	**76.3%**
Region of journal^1^							
Europe	11	2111	2008	4119	1.211 (0.922**–**1.589)	0.169	35.8%
America	26	15,362	24,250	39,612	**1.293 (1.126–1.485)**	** < 0.001**	33.2%
Asia	10	7022	7067	14,089	**0.751 (0.616–0.914)**	**0.004**	**78.2%**
No. of center							
Single center	23	3081	2641	5722	1.021 (0.772**–**1.352)	0.882	0.0%
Multi-center	25	14,898	12,852	27,750	**1.138 (1.017–1.274)**	**0.024**	**79.5%**
Surgical approach^2^							
LRH vs ARH	26	11,312	10,633	21,945	1.087 (0.956**–**1.235)	0.204	**75.5%**
RRH vs ARH	11	2217	2253	4470	1.094 (0.856**–**1.399)	0.474	**53.9%**
MIS vs ARH	14	7817	6057	13,874	1.245 (0.998**–**1.552)	0.052	46.6%
Region of center^3^							
Europe	13	1917	2113	4030	1.016 (0.710**–**1.452)	0.932	28.7%
America	12	4270	4059	8329	**1.399 (1.113–1.759)**	**0.004**	39.2%
Asia	21	13,958	11,482	25,440	1.028 (0.906**–**1.166)	0.672	**72.4%**
Sample volume^4^							
High volume	21	9111	6270	15,381	1.141 (0.963**–**1.351)	0.128	44.9%
Low volume	22	5765	5561	11,326	**1.227 (1.002–1.502)**	**0.047**	23.7%
Mixed volume	5	6269	6833	13,102	0.980 (0.849**–**1.132)	0.786	**94.5%**
MIS sample volume^4^							
High volume	22	8109	4960	13,069	1.016 (0.843**–**1.225)	0.867	6.3%
Low volume	21	6767	6871	13,638	**1.267 (1.067–1.505)**	**0.007**	42.7%
Mixed volume	5	6269	6833	13,102	0.980 (0.849**–**1.132)	0.786	**94.5%**

Data in bold style were of statistical significance to faciliate reading

*MIS* minimally invasive surgery, *LRH* laparoscopic radical hysterectomy, *RRH* robotic-assisted radical hysterectomy, *ARH* abdominal radical hysterectomy, *HR* hazard ratio, *CI* confidence interval

^1^One study published in an Australian journal was not presented in the table;

^2^Two studies reported both LRH and RRH;

^3^Two studies conducted intercontinental were not presented in the table;

^4^Sample volume and MIS sample volume were estimated by total number of patients received  radical hysterectomy by all means and by MIS before propensity scored matching dividing number of centers and years of recruitment

### Meta-analyses of LRH versus ARH and RRH versus ARH

Thirty-four studies were included for meta-analysis of DFS/PFS comparing LRH to ARH. Total number of patient before propensity scored matching was 26,886 (MIS 11270 and ARH 15252) and 17,778 patients (MIS 9284 and ARH 8494) after matching were included for meta-analysis. Overall, patients that received LRH had higher risk of recurrence than patients who received ARH (HR 1.277; 95% CI 1.143–1.426). However, stratified analyses showed comparable PFS/DFS between patients that underwent MIS and ARH in studies published before the LACC trial (HR 0.858; 95% CI 0.668–1.103), published in European (HR 0.858; 95% CI 0.640–1.151) and Asian (HR 1.105; 95% CI 0.856–1.426) journals, with a single-center study design (HR 0.996; 95% CI 0.768–1.291), conducted in centers in Europe (HR 1.226; 95% CI 0.914–1.643) or in centers with high MIS sample volume (HR 0.971; 95% CI 0.790–1.194) (Table 4).

**Table 4:** Sub-group analysis of studies comparing disease-free/progression-free survival between patients undergoing LRH and ARH

	No. of studies	No. of patients			HR (95% CI)	*p* value	*I*^2^ value
		MIS	ARH	Total			
Overall analysis	34	9284	8494	17,778	**1.277 (1.143–1.426)**	** < 0.001**	45.2%
Publication time-point							
Before LACC	17	3871	2912	6783	0.858 (0.668–1.103)	0.233	0.0%
After LACC	17	5413	5582	10,995	**1.474 (1.307–1.662)**	** < 0.001**	**55.7%**
Region of journal							
Europe	10	1731	1378	3266	0.858 (0.640–1.151)	0.306	9.9%
America	17	4331	4765	9096	**1.453 (1.278–1.652)**	** < 0.001**	46.1%
Asia	7	3508	2789	6297	1.105 (0.856–1.426)	0.444	36.0%
No. of center							
Single center	21	2607	2244	4851	0.996 (0.76﻿8﻿–1.291)	0.973	0.0%
Multi-center	13	6677	6250	12,927	**1.358 (1.202–1.535)**	** < 0.001**	**67.2%**
Region of center^1^							
Europe	10	722	891	1613	1.226 (0.914–1.643)	0.174	46.6%
America	1	–	–	–	**–**	**–**	–
Asia	22	7832	6866	14,698	**1.262 (1.115–1.428)**	** < 0.001**	**50.2%**
Sample volume^2^							
High volume	17	4760	3693	8453	1.096 (0.923–1.302)	0.296	49.0%
Low volume	17	4524	4801	9325	**1.396 (1.209–1.612)**	** < 0.001**	43.1%
MIS sample volume^2^							
High volume	17	4894	3585	8479	0.971 (0.790–1.194)	0.781	35.7%
Low volume	17	4390	4909	9299	**1.457 (1.279–1.660)**	** < 0.001**	37.6%

Data in bold style were of statistical significance to faciliate reading

*MIS* minimally invasive surgery, *LRH* laparoscopic radical hysterectomy, *RRH* robotic-assisted radical hysterectomy, *ARH* abdominal radical hysterectomy, *HR* hazard ratio, *CI* confidence interval

^1^One study was intercontinental

^2^Sample volume and MIS sample volume were estimated by total number of patients received  radical hysterectomy by all means and by MIS before propensity scored matching dividing number of centers and years of recruitment

Twenty-five studies were included for meta-analysis of OS comparing LRH to ARH. The total number of patients before propensity scored matching was 30,063 (MIS 13245 and ARH 10633), and 21,945 (MIS 11312 and ARH 10633) after matching were included for meta-analysis. Overall, there was no difference of OS between patients that underwent LRH and ARH. However, LRH was associated with a poor OS in studies published in American journals (HR 1.258; 95% CI 1.023–1.547) and conducted in centers with a low MIS sample volume (HR 1.249; 95% CI 1.007–1.550), but with a better OS in studies published in Asian journals (HR 0.718; 95% CI 0.587–0.877) (Table 5).

**Table 5:** Sub-group analysis of studies comparing overall survival between patients undergoing LRH and ARH

	No. of studies	No. of patients			HR (95% CI)	*p* value	*I*^2^ value
		LRH	ARH	Total			
Overall analysis	25	11,312	10,633	21,945	0.945 (0.818–1.091)	0.440	**66.2%**
Publication time-point							
Before LACC	11	3183	2446	5629	0.861 (0.619–1.199)	0.712	0.0%
After LACC	14	8129	8187	16,316	0.971 (0.828–1.138)	0.377	**80.5%**
Region of journal							
Europe	7	949	693	1642	0.867 (0.478–1.572)	0.638	0.0%
America	11	3769	4063	7832	**1.258 (1.023–1.547)**	**0.030**	12.6%
Asia	7	6594	5877	12,471	**0.718 (0.587–0.877)**	**0.001**	**66.3%**
No. of center							
Single center	15	1839	1695	3534	0.954 (0.642–1.420)	0.529	0.0%
Multi-center	10	6307	5767	12,074	0.942 (0.811–1.096)	0.441	**84.5%**
Region of center^1^							
Europe	6	758	334	424	0.934 (0.453–1.926)	0.853	0.0%
America	1	**–**	**–**	–	**–**	**–**	–
Asia	17	10,248	9472	19,720	0.908 (0.782–1.053)	0.848	**71.8%**
Sample volume^2^							
High volume	11	3900	3027	6927	0.916 (0.685–1.225)	0.553	32.4%
Low volume	13	4246	4435	8681	1.233 (0.990–1.536)	0.062	0.0%
Mixed volume	1	**–**	**–**	–	**–**	**–**	–
MIS sample volume^2^							
High volume	13	4091	3047	7138	0.902 (0.669–1.216)	0.497	20.1%
Low volume	11	4055	4415	8470	**1.249 (1.007–1.550)**	**0.043**	15.2%
Mixed volume	1	**–**	**–**	–	**–**	**–**	–

Data in bold style were of statistical significance to faciliate reading

*MIS* minimally invasive surgery, *LRH* laparoscopic radical hysterectomy, *RRH* robotic-assisted radical hysterectomy, *ARH* abdominal radical hysterectomy, *HR* hazard ratio, *CI* confidence interval

^1^One study was intercontinental

^2^Sample volume and MIS sample volume were estimated by total number of patients received  radical hysterectomy by all means and by MIS before propensity scored matching dividing number of centers and years of recruitment

Thirteen studies were included for meta-analysis of PFS/DFS comparing RRH to ARH. The total number of patient before propensity scored matching was 14,044 (RRH 3103 and ARH 10941), and 5084 (MIS 2534 and ARH 2550) after matching were included for meta-analysis. Patients that received RRH had higher risk of death than patients who received ARH (HR 1.303; 95% CI 1.130–1.503). However, there were comparable OS between patients that underwent MIS and ARH in studies conducted in a single center (HR 1.516; 95% CI 0.970–2.369) or in Europe (HR 1.376; 95% CI 0.940–2.014) (Table 6).

**Table 6:** Sub-group analysis of studies comparing disease-free/progression-free survival between patients undergoing RRH and ARH

	No. of studies	No. of patients			HR (95% CI)	*p* value	*I*^2^ value
		RRH	ARH	Total			
Overall analysis	13	2534	2550	5084	**1.303 (1.130–1.503)**	** < 0.001**	**57.9%**
Publication time-point							
Before LACC	7	982	1036	2018	**1.717 (1.156–2.551)**	**0.007**	0.0%
After LACC	6	1552	1514	3066	**1.244 (1.068–1.450)**	**0.005**	**55.7%**
Region of journal							
Europe	5	857	800	1657	**1.781 (1.268–2.503)**	**0.042**	**59.7%**
America	6	1418	1308	2726	**1.189 (1.015–1.394)**	**0.049**	**55.0%**
Asia	2	259	442	701	2.263 (0.989–5.177)	0.329	0.0%
No. of center							
Single center	8	906	765	1670	1.516 (0.970–2.369)	0.068	44.2%
Multi-center	5	1629	1785	3414	**1.277 (1.098–1.484)**	**0.001**	**72.5%**
Region of center^1^							
Europe	5	810	755	1565	1.376 (0.940–2.014)	0.101	**51.4%**
America	6	677	774	1451	**1.736 (1.200–2.511)**	**0.003**	34.9%
Asia	2	1047	1021	2068	**1.241 (1.049–1.467)**	**0.012**	**51.2%**
Sample volume^2^							
High volume	8	2114	1949	4063	**1.283 (1.111–1.482)**	**0.001**	**72.7%**
Low volume	5	420	601	1021	1.586 (0.795–3.166)	0.191	0.0%
MIS sample volume^2^							
High volume	7	1134	928	2062	**1.537 (1.131–2.090)**	**0.006**	**65.3%**
Low volume	6	1400	1622	3022	**1.258 (1.072–1.477)**	**0.005**	43.6%

Data in bold style were of statistical significance to faciliate reading

*MIS* minimally invasive surgery, *LRH* laparoscopic radical hysterectomy, *RRH* robotic-assisted radical hysterectomy, *ARH* abdominal radical hysterectomy, *HR* hazard ratio, *CI* confidence interval

^1^One study was intercontinental

^2^Sample volume and MIS sample volume were estimated by total number of patients received  radical hysterectomy by all means and by MIS before propensity scored matching dividing number of centers and years of recruitment

Eleven studies with 4470 patients (RRH 2217, ARH 2253) were included to evaluate OS of patients that received RRH. There was no difference of OS between RRH and ARH.

### Meta-analyses of MIS versus ARH in patients with FIGO IB1 cervical cancer

Seventeen studies with 13,944 patients after matching (MIS 7168, 6776) specifically compared patients with FIGO IB1 cervical cancer underwent MIS and ARH. Overall, MIS was associated with increased risk of recurrence and progression in patients with FIGO IB1 cervical cancer (HR 1.515; 95% CI 1.271–1.805). Subgroup analyses showed comparable OS between patients that underwent MIS and ARH in studies with a single-center design (HR 1.558; 95% CI 0.911–2.664), conducted in Europe (HR 1.241; 95% CI 0.891–1.728) and with high sample volume (HR 1.254; 95% CI 0.862–1.824) or high MIS sample volume (HR 1.264; 95% CI 0.876–1.824) (Table 7). Moreover, MIS was correlated with increased risk of recurrence in patients with tumor size ≥ 2 cm (HR 1.787; 95% CI 1.396–2.286) but not < 2 cm (HR 1.257; 95% CI 0.884–1.789). While in 8375 FIGO IB1 patients with tumor < 2 cm (MIS 4333, ARH 4042), although overall meta-analysis showed comparable PFS/DFS between patients that underwent MIS and ARH, MIS was correlated with increased risk of recurrence in studies conducted in Asia (HR 1.398; 95% CI 1.061–1.843) and in studies with low sample volume (HR 1.552; 95% CI 1.190–2.024) or low MIS sample volume (HR 1.527; 95% CI 1.183–1.969) (Table 8).

**Table 7:** Sub-group analysis of studies comparing disease-free survival/progression-free survival between FIGO IB1 patients undergoing MIS and ARH

	No. of studies	No. of patients			HR (95% CI)	*p* value	*I*^2^ value
		MIS	ARH	Total			
Overall analysis	17	7168	6776	13,944	**1.515 (1.271–1.805)**	** < 0.001**	38.8%
Tumor size							
IB1 < 2 cm	13	4333	4042	8375	1.257 (0.884–1.789)	0.311	48.5%
IB1 ≥ 2 cm	9	2201	2055	4256	**1.787 (1.396–2.286)**	**0.025**	14.7%
Region of center^1^							
Europe	4	776	1199	1975	1.241 (0.891–1.728)	0.202	**66.4%**
America	3	376	243	619	**2.478 (1.148–5.348)**	**0.021**	**58.8%**
Asia	9	5109	4452	9561	**1.523 (1.213–1.912)**	** < 0.001**	28.7%
Surgical approach							
LRH vs ARH	9	4114	4368	8482	**1.575 (1.277–1.943)**	** < 0.001**	44.2%
RRH vs ARH	3	540	547	1087	1.502 (0.729–3.095)	0.270	5.1%
MIS vs ARH	6	2274	1659	3933	1.392 (0.994–1.950)	0.054	**53.3%**
No. of centers							
Single-center	3	222	201	423	1.558 (0.911–2.664)	0.105	45.9%
Multi-center	14	6706	6373	13,079	**1.513 (1.264–1.812)**	** < 0.001**	40.1%
Sample volume^2^							
High volume	4	2411	1523	3934	1.254 (0.862–1.824)	0.236	38.6%
Low volume	11	3947	4069	8016	**1.739 (1.397–2.165)**	** < 0.001**	27.4%
Mixed volume	2	570	982	1552	1.151 (0.783–1.692)	0.720	**81.3%**
MIS sample volume^2^							
High volume	5	2093	1177	3270	1.264 (0.876–1.824)	0.211	47.3%
Low volume	10	4415	4265	8680	**1.693 (1.354–2.118)**	** < 0.001**	14.4%
Mixed volume	2	570	982	1552	1.151 (0.783–1.692)	0.474	**81.3%**

Data in bold style were of statistical significance to faciliate reading

*MIS* minimally invasive surgery, *LRH* laparoscopic radical hysterectomy, *RRH* robotic-assisted radical hysterectomy, *ARH* abdominal radical hysterectomy, *HR* hazard ratio, *CI* confidence interval

^1^One study was intercontinental

^2^Sample volume and MIS sample volume were estimated by total number of patients received  radical hysterectomy by all means and by MIS before propensity scored matching dividing number of centers and years of recruitment

**Table 8:** Sub-group analysis of studies comparing disease-free survival/progression-free survival between FIGO IB1 patients with tumor size < 2 cm undergoing MIS and ARH

	No. of studies	No. of patients			HR (95% CI)	*p* value	*I*^2^ value
		MIS	ARH	Total			
FIGO IB1 < 2 cm	13	4333	4042	8375	1.257 (0.884–1.789)	0.311	48.5%
Region of center^1^							
Europe	2	320	365	685	**0.516 (0.281–0.945)**	**0.032**	0.0%
America	2	212	103	315	3.569 (0.990–12.867)	0.052	20.4%
Asia	8	3315	3082	6397	**1.398 (1.061–1.843)**	0.017	31.5%
Surgical approach							
LRH vs ARH	8	2748	2961	5709	**1.291 (1.007–1.655)**	**0.044**	**54.8%**
RRH vs ARH	2	521	512	1033	1.285 (0.565–2.924)	0.550	0.0%
MIS vs ARH	3	1064	569	1633	1.075 (0.562–2.059)	0.826	73.4%
Sample volume^2^							
High volume	4	1474	988	2462	0.852 (0.543–1.338)	0.487	0.0%
Low volume	8	2676	2803	5479	**1.552 (1.190–2.024)**	**0.001**	38.5%
Mixed volume	1	183	251	434	0.440 (0.160–1.270)	–	**–**
MIS sample volume^2^							
High volume	3	983	497	1480	0.745 (0.442–1.255)	0.269	0.0%
Low volume	9	3167	3294	6461	**1.527 (1.183–1.969)**	**0.001**	30.9%
Mixed volume	1	183	251	434	0.440 (0.160–1.270)	–	**–**

Data in bold style were of statistical significance to faciliate reading

*MIS* minimally invasive surgery, *LRH* laparoscopic radical hysterectomy, *RRH* robotic-assisted radical hysterectomy, *ARH* abdominal radical hysterectomy, *HR* hazard ratio, *CI* confidence interval

^1^One study was intercontinental

^2^Sample volume and MIS sample volume were estimated by total number of patients received  radical hysterectomy by all means and by MIS before propensity scored matching dividing number of centers and years of recruitment

### Publication bias

**Fig. 2:**
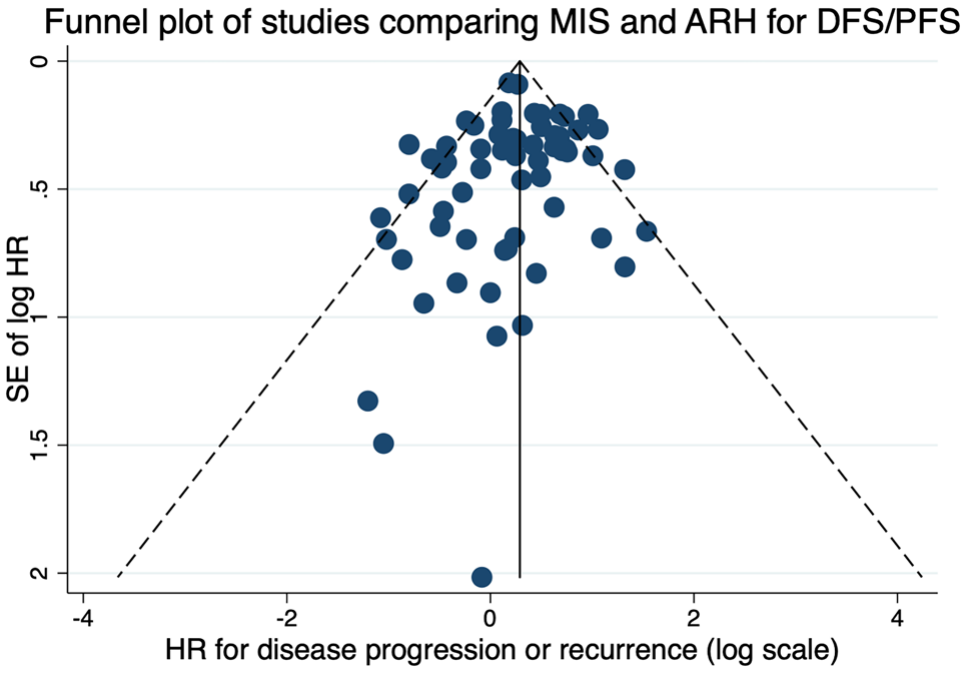
Funnel plots for studies comparing disease-free survival/progression-free survival

**Fig. 3:**
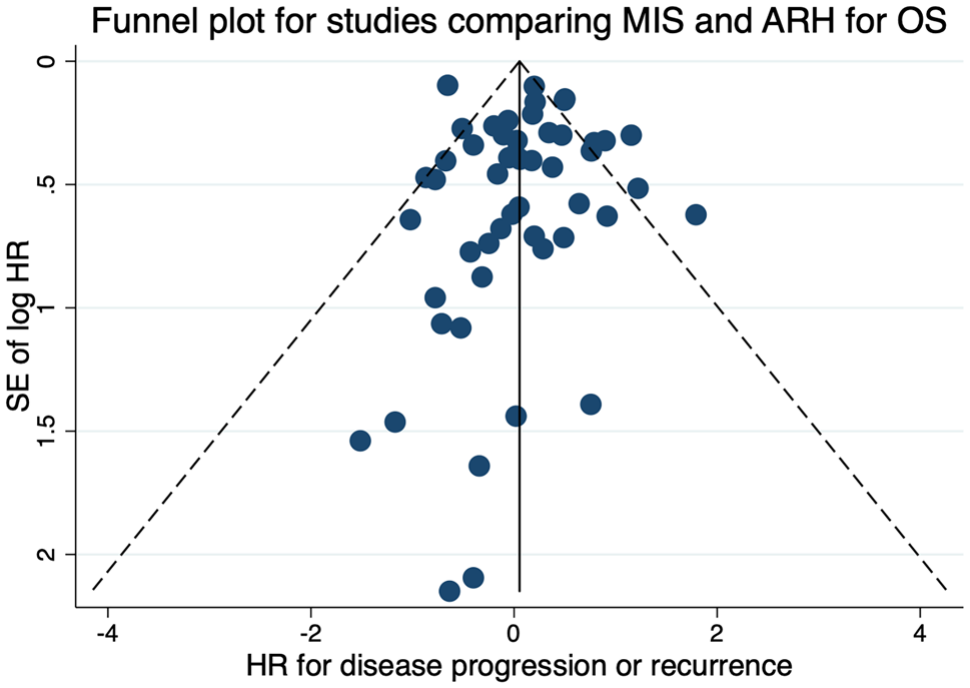
Funnel plots for studies comparing overall survival

Publication bias was first evaluated by visual inspection of funnel plots and then Egger’s test. Visual inspection of funnel plots for studies comparing DFS/PFS and OS of patients undergoing MIS and ARH showed a slight asymmetry (Figs. 2 and 3). The results of Egger’s tests suggested that there was no publication bias for pooled DFS/PFS (*p* = 0.074) and OS (*p* = 0.052). Sensitivity analysis was performed by sequentially trimming and adding each included study. The results remained unchanged.

## Discussion

Overall, compared to ARH, MIS was associated with increased risk of disease progression or recurrence and increased risk of death in women with early stage cervical cancer. Comparable oncological outcomes between patients that received MIS and ARH was found in the meta-analysis in FIGO IB1 patients with tumor size less than 2 cm and in studies published before the LACC trial, published in European journals, conducted in a single center, performed in centers in Europe or with a high MIS sample volume, while the inferiority of MIS was found in the meta-analysis of studies published after the LACC trial, with a multi-center study design, conducted in Asia and America, or in centers with a low MIS sample volume. These findings delineate the complexity of the factors impacting MIS outcomes reported in published studies and may trigger rethinking about the surgical approaches for radical hysterectomy in early stage cervical cancers.

We found comparable oncological safety in patients undergoing MIS compared with ARH in studies published before the LACC trial but inferiority of MIS in studies published after the LACC trial, which was consistent with previous meta-analyses [9, 10, 15–17]. Additionally, studies published in American journals showed a poor PFS in patients that received MIS while studies published in European and Asian journals showed comparable PFS between patients undergoing MIS and ARH. We assumed that different characteristics of publication such as year and journal of publication might be a reason that led to the heterogeneous results in this study. The comparison between MIS to ARH also revealed magnificent geographical difference. Namely, studies in Asia reported that both LRH and RRH were associated with increased risk of recurrence and progression in patients with cervical cancer, which was consistent with the results reported by Hwang et al. [17]. Studies in America reported poor PFS in patients that received MIS except those with FIGO IB1 disease while studies in Europe reported comparable DFS/PFS between MIS and ARH. Regional subgroup analyses revealed high consistency in Europe but marked heterogeneity in Asia and America. Based on the present study, we did not find any evidence opposing MIS as an alternative choice of ARH in Europe. However, the results of our study as well as most previous meta-analyses were based primarily on non-randomized studies and should be, therefore, interpreted as generating hypotheses.

Comparable oncological safeties of MIS vs. ARH and LRH vs. ARH were observed in centers with a high sample volume or high MIS sample volume but poor outcome of MIS and LRH in centers with a low sample or low MIS sample volume. A retrospective analysis involving 116 Japanese centers, where 5964 women with FIGO IB1-IIB cervical cancer underwent radical hysterectomy, revealed a significantly decreased risk for recurrence (HR 0.69; 95% CI 0.57–0.84) and death (HR 0.75; 95% CI 0.59–0.95) in high-volume centers when compared with low-volume centers [74]. According to data reported by Matsuo et al., a population-based retrospective study queried the American National Inpatient Sample from 2007 to 2011, the centers favoring RRH were more likely to be small bed-capacity hospitals and less likely to be urban-teaching hospitals [75]. In this case, instead of being a reflection of proficiency of treatment centers, what higher RRH volume represented was just the other way around. These findings implied that whether MIS was comparable to ARH was center-associated, which was consistent with the findings by Gennari et al. that the treatment center remained a strong prognostic factor regarding recurrence-free survival (RFS) (high-volume vs. low-volume HR 0.49; 95% CI 0.28–0.83) and OS (high-volume vs. low-volume HR 0.50; 95% CI, 0.26–0.94) [29].

MIS was associated with increased risk of recurrence and progression in studies with a multi-center design but not in studies with a single center design. This difference might be partially due to the variances of centers involved in multi-center studies and single-center studies: ideally, in a single-center study, the center should be capable of providing sufficient number of MIS cases, while in a multi-center study, the centers with low sample volume should be limited. However, there was disproportion between number of included centers and number of patients as well as mixture of centers with different sample volume, bed capacity and different MIS technique level in several intercontinental, nation-wide and vast regional multi-center studies. Additionally, some local high-volume centers were not readily included in multi-center studies from the same region. For example, centers from studies reported by He et al. [60] and Yang et al. [5] were absent in the study reported by Chen et al. [40]. The absence of the local high-volume centers in multi-center studies could further augment the difference between the single-center studies and the multi-center studies and led to results favoring ARH. Compared to assessment of medical therapies, the assessment of surgical approaches was even more difficult due to heterogeneities of personal skills, surgical instruments, experiences of surgical team, supportive medication of complication and adjuvant therapy, let alone a surgery as challenging as minimally invasive radical hysterectomy. Even RCTs, the most rigorous study design, were difficult to conduct rigorously in the evaluation of some complex surgical interventions [76]. Therefore, the reported increased risk of recurrence of cervical cancer associated with MIS might reflect the uneven proficiency in the MIS technique around the world rather than the inferiority of the surgical approach itself [77, 78]. Center-associated factors such as center sample volume and experience of surgeons needed to be taken consideration in future evaluation of MIS hysterectomy.

Study inclusion was maximized and subgroup analyses based on characteristics of disease, publication, study design and treatment center were performed so as to get a general idea of actual oncological safety of MIS for cervical cancer among previous heterogeneous results. Meanwhile, several limitations came along with this study design. A few earlier studies did not report adjusting method for variable control and the cohort scale of these earlier studies was also relatively smaller as compared to that of recent multicenter studies. The sample volume and MIS sample volume for multi-center studies might not represent the actual surgical volume of each included center since most multi-center studies did not report the exact number of included patients from each center. We did not evaluate the potential impact of protective maneuvers on improving the oncological safety of the laparoscopic radical hysterectomy technique as suggested by Kampers et al. [79]. And we failed to perform stratified analyses based on the proficiency of the treatment center and the MIS technique of the surgeons.

## Conclusions

Our findings highlight possible factors that contributed to inferior performance of MIS in cervical cancer including publication characteristics, center-geography and sample volume. Center associated factors were needed to be taken into consideration when evaluating complex surgical procedures like radical hysterectomy.

## Supplementary Information

Below is the link to the electronic supplementary material.

Supplementary file1 (DOCX 21 KB)

Supplementary file2 (XLSX 23 KB)
