# Supplementary material for: A meta-analysis of survival after minimally invasive radical hysterectomy versus abdominal radical hysterectomy in cervical cancer: center-associated factors matter
Source: Arch Gynecol Obstet. 2022 Jan 21;306(3):623–37. doi: 10.1007/s00404-021-06348-5 (PMC9411220; doi:10.1007/s00404-021-06348-5)
Supplement: Supplementary file 1 — Supplementary file1 (DOCX 21 KB) [file 404_2021_6348_MOESM1_ESM.docx]

**Method of quality assessment for the included studies.**

NEWCASTLE - OTTAWA QUALITY ASSESSMENT SCALE COHORT STUDIES

SELECTION

1. Representativeness of the Exposed Cohort

2) Selection of the Non-Exposed Cohort

3) Ascertainment of Exposure

4) Demonstration That Outcome of Interest Was Not Present at Start of Study

COMPARABILITY

1) Comparability of Cohorts on the Basis of the Design or Analysis

OUTCOME

1) Assessment of Outcome For some outcomes (e.g. fractured hip), reference to the medical record is sufficient to satisfy the requirement for confirmation of the fracture. This would not be adequate for vertebral fracture outcomes where reference to x-rays would be required.

a) Independent or blind assessment stated in the paper, or confirmation of the outcome by reference to secure records (x-rays, medical records, etc.)

b) Record linkage (e.g. identified through ICD codes on database records)

c) Self-report (i.e. no reference to original medical records or x-rays to confirm the outcome)

d) No description.

2) Was Follow-Up Long Enough for Outcomes to Occur An acceptable length of time should be decided before quality assessment begins (e.g. 5 yrs. for exposure to breast implants)

3) Adequacy of Follow Up of Cohorts This item assesses the follow-up of the exposed and non-exposed cohorts to ensure that losses are not related to either the exposure or the outcome. Allocation of stars as per rating sheet

**List of scores for included studies.**

| **Author** | **Year of publication** | **NOS score** |
| --- | --- | --- |
| Gennari P | 2021 | 7 |
| Kim S | 2021 | 6 |
| Levine M | 2021 | 7 |
| Li L | 2021 | 6 |
| Rodriguez J | 2021 | 7 |
| Zaccarini F | 2021 | 6 |
| Bogani G | 2020 | 6 |
| Brandt B | 2020 | 7 |
| Chen B | 2020 | 7 |
| Chen C | 2020 | 7 |
| Chen X | 2020 | 7 |
| Chiva L | 2020 | 7 |
| Dai D | 2020 | 7 |
| Eoh K | 2020 | 6 |
| Guo C | 2020 | 7 |
| He J | 2020 | 7 |
| Hu T | 2020 | 7 |
| Pedone L | 2020 | 6 |
| Qin M | 2020 | 7 |
| Uppal S | 2020 | 7 |
| Wenzel H | 2020 | 7 |
| Yang J | 2020 | 7 |
| Yang W | 2020 | 7 |
| Yuce T | 2020 | 6 |
| Alfonzo E | 2019 | 8 |
| Cusimano M | 2019 | 7 |
| Doo D | 2019 | 7 |
| Gil-Moreno A | 2019 | 7 |
| Kanao H | 2019 | 5 |
| Kim J | 2019 | 6 |
| Kim S | 2019 | 7 |
| Liu Y | 2019 | 6 |
| Matanes E | 2019 | 6 |
| Paik E | 2019 | 7 |
| Ratiu D | 2019 | 7 |
| Yuan Z | 2019 | 8 |
| Melamed A | 2018 | 7 |
| Corrado G | 2018 | 6 |
| Guo J | 2018 | 5 |
| Pedro R | 2018 | 6 |
| Driver | 2017 | 6 |
| He H | 2017 | 7 |
| Shah C | 2017 | 7 |
| Wallin E | 2017 | 7 |
| Mendivil A | 2016 | 6 |
| Sert B | 2016 | 5 |
| Wang W | 2016 | 8 |
| Zanagnolo V | 2016 | 5 |
| Ditto A | 2015 | 8 |
| Xiao M | 2015 | 5 |
| Yang L | 2015 | 6 |
| Bogani G | 2014 | 5 |
| Toptas T | 2014 | 6 |
| Park J | 2013 | 5 |
| Choi C | 2012 | 5 |
| Nam J | 2012 | 8 |
| Lee E | 2011 | 5 |
| Yang Z | 2011 | 6 |
| Cantrell L | 2010 | 5 |
| Malzoni M | 2009 | 5 |
| Sobiczewski P | 2009 | 5 |
